# A Systematic Review of Interventions That Integrate Perinatal Mental Health Care Into Routine Maternal Care in Low- and Middle-Income Countries

**DOI:** 10.3389/fpsyt.2022.859341

**Published:** 2022-03-14

**Authors:** Maria C. Prom, Amrutha Denduluri, Lisa L. Philpotts, Marta B. Rondon, Christina P. C. Borba, Bizu Gelaye, Nancy Byatt

**Affiliations:** ^1^Chester M. Pierce Division of Global Psychiatry, Massachusetts General Hospital, Harvard Medical School, Boston, MA, United States; ^2^Department of Epidemiology, Harvard T.H. Chan School of Public Health, Boston, MA, United States; ^3^Treadwell Library, Massachusetts General Hospital, Boston, MA, United States; ^4^Department of Psychiatry, Instituto Nacional Materno Perinatal, Lima, Peru; ^5^Department of Psychiatry, Global and Local Center for Mental Health Disparities, Boston Medical Center, Boston University School of Medicine, Boston, MA, United States; ^6^Department of Psychiatry, University of Massachusetts Chan Medical School, UMass Memorial Health Care, Worcester, MA, United States

**Keywords:** mental health, perinatal anxiety, perinatal depression, systematic review, low- and middle-income countries (LMICs), integrated care

## Abstract

**Background:**

Women in low- and middle-income countries (LMICs) are disproportionally affected by perinatal depression and anxiety and lack access to mental health care. Integrating perinatal mental health care into routine maternal care is recommended to address gaps in access to mental health care in such under-resourced settings. Understanding the effectiveness of interventions that integrate perinatal mental health care into routine maternal care in LMICs is critical to inform ongoing intervention development, implementation, and scale-up. This systematic review aims to assess the effectiveness of interventions that integrate perinatal mental health care into routine maternal care to improve maternal mental health and infant health outcomes in LMICs.

**Method:**

In accordance with the PRISMA guidelines, an electronic database search was conducted seeking publications of controlled trials examining interventions that aimed to integrate perinatal mental health care into routine maternal care in LMICs. Abstracts and full text articles were independently reviewed by two authors for inclusion utilizing Covidence Review Software. Data was extracted and narrative synthesis was conducted.

**Findings:**

Twenty studies met eligibility criteria from the initial search results of 2,382 unique citations. There was substantial heterogeneity between the study samples, intervention designs, and outcome assessments. Less than half of the studies focused on women with active depression or anxiety. Most studies (85%) implemented single intervention designs involving psychological, psychosocial, psychoeducational, or adjuvant emotion/stress management. There were few interventions utilizing multicomponent approaches, pharmacotherapy, or referral to mental health specialists. Outcome measures and assessment timing were highly variable. Eighteen studies demonstrated significantly greater improvement on depression and/or anxiety measures in the intervention group(s) as compared to control.

**Conclusion:**

Integrated interventions can be effective in LMICs. The findings provide a critical understanding of current interventions design gaps. This includes the lack of comprehensive intervention designs that incorporate increasing intensity of treatment for more severe illness, pharmacotherapy, mental health specialist referrals, and non-mental health professional training and supervision. The findings also provide strategies to overcome design and implementation barriers in LMICs. Study findings provide a foundation for future evidence-based adaptation, implementation, and scale-up of interventions that integrate perinatal mental health care into routine maternal care in LMICs.

**Systematic Review Registration:**

[https://www.crd.york.ac.uk/prospero/display_ record.php?ID=CRD42021259092], identifier [CRD42021259092].

## Introduction

Mental health and substance use disorders are the leading causes of morbidity, accounting for 22.8% of global years of healthy life lost due to disability (YLDs) (1). The majority of that burden exists in low-and middle-income countries (LMICs) (2, 3), impacting both individuals and their families. This results in economic and social hardships that affect society as a whole, furthering the cycle of poverty and health inequities.

Women with perinatal depression and anxiety disorders, occurring during pregnancy and 1 year after delivery, are at increased risk of obstetric complications and poor infant outcomes. This includes low birth weight, pre-eclampsia, pre-term delivery, inadequate perinatal care, poor nutrition, increased substance use, suicide, disruption of maternal-infant bonding and attachment, and in severe cases, infanticide. Offspring are at further risk of behavioral, emotional, cognitive, language and motor development challenges, with current evidence spanning from infancy through adolescence (4–19).

As is the case for mental illness globally, the burden of perinatal depression and anxiety is greater in LMICs than high-income countries (HICs). The estimated prevalence of perinatal depression in LMICs is 25% in the antepartum period vs. 7–15% in HICs and 20% in the postpartum period vs. 10% in HICs (5, 20–25). The estimated prevalence of antepartum anxiety in LMICs is 18% in the antepartum period and 13% in HICs (26). Women who reside in LMICs are disproportionately affected due to increased exposure to risk factors, including lower socioeconomic status, early life abuse, exposure to intimate partner violence, low education levels, unintended pregnancy, and limited social support (24, 27–34). Untreated pre-existing depression contributes to the higher prevalence in the perinatal period (35). Despite the increased burden of illness for pregnant women in LMICs, there is very limited access to mental health care and an immense inequity in access as compared to HICs. For instance, there are 0.9–14.7 mental health workers per 100,000 population in LMICs as compared to 62.2 per 100,000 in HICs (36).

Addressing the lack of mental health care access in under-resourced LMICs is essential to improving maternal and infant health and interrupting the pattern of intergenerational mental illness and suffering. Given the significant lack of mental health care providers and national resources dedicated to mental health in LMICs, traditional mental health care models are insufficient to address this need. To address this, evidence-based strategies have been developed, including integrating perinatal mental health care into routine maternal care.

Integrated care interventions are a World Health Organization (WHO) recommendation for improving access in LMICs, particularly as routine maternal care can be one of the few times women in LMICs access health care (37). In HICs there is strong evidence for integrating perinatal mental health care into routine maternal care to address care access gaps in under-resourced settings (38–43). There are many existing models and interventions designed for integrating mental health care into routine medical care. These integrated care models vary significantly in their settings, components, and providers. They can range from mental health providers imbedded in clinics to training of existing medical providers to manage mental health care with referral to higher levels of specialized care if needed (such as stepped care) vs. having a care manager and psychiatrist guiding providers (such as the collaborative care model). In HICs, these integrated care models in primary care settings have demonstrated improvement in social role function, quality of life, treatment adherence, and symptom remission and recovery, while also being cost-effective (44–46). As such, there is increasing advocacy to implement programs in LMICs that integrate perinatal mental health care into maternal and child health care programs (47, 48).

Implementing evidence based integrated mental health care models developed in HICs may present a challenge in under-resourced LMIC health care systems. Such care models necessitate additional resources for implementation, such as case managers and social workers. They also often require the introduction of mental health screening and training and supervision of non-mental health professionals. Furthermore, the cultural and language differences in LMICs introduce additional barriers to implementing interventions developed in HICs. Ultimately, understanding the effectiveness of interventions that integrate perinatal mental health care into routine maternal care within LMICs is critical prior to larger implementation and scale-up. Unfortunately, there is a lack of systematic evaluation of interventions that integrate perinatal mental health care into routine maternal care in LMICs (49). This is in part a reflection of limited research conducted in LMICs, with three times more mental health research output from high-income countries than low-income countries (36). Previous systematic assessments of interventions focused on perinatal mental health care in LMICs have examined non-mental health professional task-sharing for care delivery and psychological and psychosocial interventions (50–56). However, no systematic review to this point has examined the effectiveness of interventions that integrate perinatal mental health care into routine maternal care in LMICs.

This systematic review aims to assess the effectiveness of interventions that integrate perinatal mental health care focused on depression and anxiety into routine maternal care to improve maternal mental health and infant health outcomes in LMICs as compared to usual care.

## Methods

### Systematic Review Protocol

This systematic review was conducted in accordance with the Preferred Reporting for Systematic Reviews and Meta-Analyses (PRISMA) guidelines (57). The Population Intervention Comparison Outcome (PICO) method (58) was utilized to develop the review protocol prior to conducting the database search. The protocol was registered on the PROSPERO database (59).

#### Search Strategy

The search strategy was iteratively developed with a health sciences librarian (LLP) who conducted searches for articles published up to June 2021 without language restrictions using electronic databases Medline, Cochrane CENTRAL, PsycINFO, Web of Science, EMBASE and the Global Index Medicus. Customized search strategies were developed for each database and keywords and subject headings were derived from five major concepts (1) low- and middle- income country [as classified by the World Bank (60)], (2) perinatal (3) mental health, (4) mental health services, and (5) integrated health care. Terms were adapted for use in each database. A full description of the search strategy can be found in the PROSPERO registry (59).

#### Inclusion and Exclusion Criteria

Inclusion criteria consisted of: (1) Population: women in the perinatal period [antepartum to postpartum (up to 1 year after delivery)] in low- and middle-incomes countries [per World Bank Classification (60)], (2) Intervention: perinatal mental health interventions that focus on depression or anxiety symptoms AND are integrated into routine outpatient clinical care (e.g., primary care, maternal care, pediatric care, and HIV care) (3) Comparators: usual care or enhanced usual care, (4) Outcomes: primary- changes in depression and/or anxiety score, secondary (if assessed)- birth outcomes, infant outcomes, quality of life or assessment of function, social support, and (5) Study Design: controlled trials. Exclusion criteria were: (1) women with mental illnesses other than depression or anxiety, including depression w/psychotic features, bipolar disorder, substance use disorders, primary psychotic disorders, and eating disorders, (2) qualitative studies, protocol papers, conference proceedings, editorials, non-peer reviewed articles, observational studies, (3) pharmaceutical clinical trials, and (4) labor and delivery interventions. Minor adjustments were made to the inclusion and exclusion criteria in the PROSPERO registered protocol during article review to broaden article inclusion appropriate to the systematic review aim. These adjustments included removing the population inclusion criteria of formal diagnosis of depression or anxiety and removal of exclusion criteria of provision of care by mental health professionals.

### Study Selection and Data Extraction

Article abstracts were uploaded and reviewed utilizing the Covidence Review Software. Duplicate articles were removed, and abstracts were independently reviewed by the first two authors for eligibility with reference to the inclusion and exclusion criteria. Articles meetings eligibility criteria and those that were inconclusive from the abstract review were retrieved for full text review. Authors were contacted for full-text articles that were not retrievable online. Full text articles were then independently reviewed by the first two authors for eligibility. While all abstracts were available in English, full text retrievable article language other than English included Spanish and Portuguese. Spanish and Portuguese language articles were reviewed for inclusion by the first author with secondary consensus from the second author via verbal translation of inclusion and exclusion criteria. Abstract and full text article inclusion and exclusion conflicts were resolved through discussion between the two reviewers referring to inclusion and exclusion criteria and operational definitions of criteria concepts. Data was extracted by the first and second authors utilizing a standardized data extraction form. Extracted data included study location and design, sample characteristics, control group, disorder of focus, intervention, intervention clinical setting, intervention duration/timing, intervention personnel, timing of assessment, primary and secondary outcomes/measures, and key findings.

### Synthesis of Included Studies and Analyses

Study data was synthesized utilizing narrative synthesis. Studies were summarized and comparisons were made among study setting, sample, intervention design, outcome measurement, and key findings. Due to the heterogeneity of the included studies (including selected samples, intervention design, and study outcomes), a meta-analysis was not conducted.

### Study Quality Assessment

Assessment of study quality was completed using the Downs and Black Checklist (61) which can be used to examine randomized and non-randomized studies. Study reporting, validity, bias, confounding, and power are assessed using a 27-item checklist with a maximum score of 32. The final item on the checklist, a five-point item evaluating sample size and power was dichotomized to indicate if the study reported *a priori* power and sample size calculations, for an adjusted maximum score of 28. A quality score was calculated for each article by dividing the total score by the maximum possible score.

## Results

**FIGURE 1:**
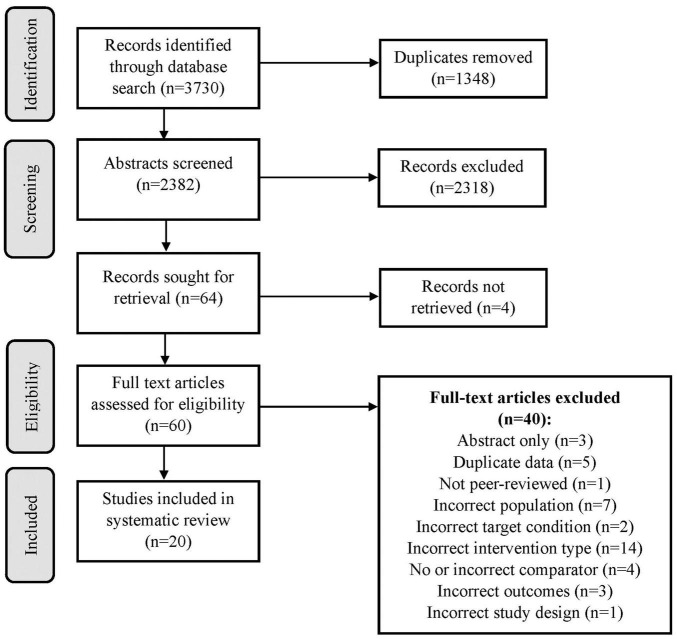
PRISMA flow diagram.

After removing duplicate results, the initial search revealed 2382 unique citations that were screened for eligibility. Abstract review resulted in exclusion of 2318 records that did not meet inclusion criteria. Full text was not retrievable for 4 records. Full text review was completed on 60 articles and 40 were excluded for not meeting inclusion criteria. Reasons for full text exclusion can be found in the flow diagram in Figure 1. Data extraction was completed on the 20 studies that met eligibility criteria.

### Study Characteristics

#### Location

All 20 studies were conducted in World Bank classified (60) middle-income countries: Chile (62), China (63–65), Iran (66–74), Kenya (75, 76), Mexico (77), Nigeria (78), Pakistan (79), and South Africa (80, 81) (Table 1). Chile is now classified by the World Bank as a high-income country but was classified as middle-income at the time of the study.

**TABLE 1:** Summary of studies selected for systematic review.

Study	Country	Study design	Sample	Comparison group	Intervention	Setting	Intervention personnel	Intervention timing; duration	Disorder of focus	Timing of assessments	Primary outcome(s)	Key Findings
												
Asadzadeh et al. (66)	Iran	Individual RCT	90 pp women who experienced a traumatic childbirth; IG *n* = 45, CG *n* = 45	Usual care	Midwife-led brief in-person and phone counseling sessions	3 governmental prenatal clinics	Midwife (clinic and research personnel)	Postpartum; 4–6 weeks (2 sessions)	Depression, anxiety, PTSD	4–6 weeks and 3 months pp	Depression (EPDS), anxiety (HAM-A), and PTSD (PCL-5)	Significantly more improvement in IG on PCL-5, EPDS, and HAM-A at 3 months follow-up.
Bastani et al. (67)	Iran	Individual RCT	110 pregnant women (14–28 weeks GA) with anxiety STAI > 20; IG *n* = 55, CG *n* = 55	Usual care	Applied relaxation training	3 prenatal clinics	Not specified	Prenatal; 7 weeks (7 sessions)	Anxiety (and stress)	1 week post-intervention	Anxiety (STAI) and perceived stress (PSS)	Significantly greater improvement in IG on STAI and PSS.
Esfandiari et al. (68)	Iran	Individual RCT	80 pregnant women (6 to 32 weeks GA); IG *n* = 40, CG *n* = 40	Usual care	Group supportive-based pregnancy stress counseling (SBPSC)	2 public health centers	Psychotherapist (research personnel)	Prenatal; 6 weeks (6 sessions)	Anxiety (and stress)	6 weeks post-intervention	Stress (NuPDQ), Anxiety (STAI-Y), Prenatal Health behaviors Scale (PHBS)	Significantly greater improvement in IG on NuPDQ, STAI-Y, PHBS, and PSS-14. No differences in cortisol.
Futterman et al. (81)	South Africa	Pilot Cluster RCT	160 pregnant women (mean 6.5 months GA) living with HIV; IG *n* = 83, CG *n* = 77	Usual care	Mentor mother support and adapted Cognitive Behavioral Intervention	1 prenatal clinic and 1 general health center	Peer mentor mothers	Prenatal; 8 sessions	Perinatal depression	6 months pp	Depression scale (CES-D), transmission risk behavior, social support, coping (COPE), infant interaction and bonding, and HIV knowledge	Significantly greater improvement in IG on CES-D, social support, and increased knowledge about HIV/AIDS.
Gureje et al. (78)	Nigeria	Cluster RCT	686 pregnant women (16–20 weeks GA) with MDD via EPDS ≥ 12 and CIDI confirmed; IG *n* = 452; CG *n* = 234	Enhanced care [low-intensity treatment (LIT) per mhGAP Intervention Guide]	Stepped care high-intensity treatment (HIT): PST, parenting skills training, pharmacotherapy	29 governmental maternal and child care clinics	Primary maternal care provider with supervision of primary care physician (clinic personnel)	Prenatal and postpartum; 8–16 weeks total (8–16 sessions)	Perinatal depression	6 and 12 months pp	Remission of depression (EPDS < 6)	Depression remission rates: 70% in IG and 66% in CG. IG more effective for severe depression and higher rate of exclusive breastfeeding. No differences in infant outcomes and cost-effectiveness.
Jabbari et al. (69)	Iran	Individual RCT	168 pregnant women (25–28 weeks GA); IG 1 *n* = 55, IG 2 *n* = 57, CG *n* = 56	Usual care	Listening to a recording of the Quran (IG 1) with translation or (IG 2) without translation	Health centers and home	Not specified	Prenatal; 3 weeks	Anxiety, perinatal depression (and stress)	4 and 8 weeks post-intervention	Perinatal depression (EPDS), Anxiety (STAI), Stress (PSS)	Significantly greater improvement in both IGs on EPDS, STAI, and PSS.
Jannati et al. (70)	Iran	Individual RCT	75 pp women (within 6 months) with PPD (EPDS ≥ 13 and MINI confirmed), IG *n* = 38, CG *n* = 37	Usual care	Mobile phone application-based CBT	Home (recruiter from health centers)	Mobile application	Postpartum; 8 weeks (8 lessons)	PPD	Immediate post-intervention	PPD (EPDS)	Significantly greater improvement in IG on EPDS
Kariuki et al. (76)	Kenya	Quasi- cluster RCT	567 pp women (6–10 weeks); IG *n* = 284, CG *n* = 283	Usual care	Psychoeducational intervention focused on PPD, coping skills, mother/child interaction and infant stimulation	2 Maternal and child health clinics	Community health nurses (research personnel)	Postpartum; 1 session	PPD	6 months post-intervention	PPD (BDI)	Significantly greater decrease in IG on BDI.
Lara et al. (77)	Mexico	Individual RCT	377 pregnant women (1st and 2nd trimester) at high risk for depression (CES-D ≥ 16 and/or history of depression); IG *n* = 250, CG *n* = 127	Usual care	Group manualized psychoeducational intervention for perinatal depression	3 health institutions: perinatal hospital high-risk pregnancy clinic, Armed forces women’s clinic, community health care center	‘Facilitators’ with clinical experience	Prenatal; 8 weeks	Perinatal depression and anxiety	6 weeks and 4–6 months pp	Perinatal depression (SCID and BDI-II)	Significantly fewer new depression cases in the IG on SCID. Significant reduction of BDI-II in IG and CG and no significant treatment effect.
Mao et al. (63)	China	Individual RCT	240 pregnant women (32 weeks GA); IG *n* = 120, CG *n* = 120	Usual Care (Standard antenatal education session)	Antenatal emotional self-management training program with group and individual counseling sessions	Hospital perinatal clinic	Obstetrician (research personnel)	Prenatal; 4 weeks (4 sessions)	Perinatal depression	Immediate post-intervention and 6 weeks pp	PPD (PHQ-9, EPDS and SCID)	Significantly greater improvement in IG on PHQ-9 and EPDS and fewer diagnoses of PPD (SCID).
Mohammadi et al. (71)	Iran	Individual RCT	127 pregnant women (26–32 weeks GA); IG 1 *n* = 43, IG 2 *n* = 42, CG *n* = 42	Usual care	Exercise educational session and CD, exercise regimen 20–30 min 3 times/week until delivery (IG 1) or 2 months pp (IG 2)	14 general public health centers with enhanced prenatal care and home	Not specified	Prenatal and postpartum; 8–22 weeks	PPD	1 and 2 months pp	PPD (EPDS) and Fatigue (FIF)	No significant difference in change in EPDS or Fatigue scores in IG and CG.
Mutisya et al. (75)	Kenya	Quasi-cluster RCT	288 pregnant women (1st and 2nd trimester) with GBV; IG *n* = 144, CG *n* = 144	Usual care	Psychosocial support sessions primarily focused on GBV	12 public primary health care facilities	Research assistants experienced in social work	Prenatal; 4–5 months (at least 3 sessions)	Perinatal depression	Immediate post-intervention	Perinatal depression (EPDS), GBV (AAS)	Significantly greater improvement in IG on EPDS and lower total GBV.
Nasiri et al. (72)	Iran	Individual RCT	120 pp women (3 weeks) with depression (EPDS ≥ 10, BDI-II 14–28 and interview confirmed); IG 1 *n* = 40, IG 2 *n* = 40, CG *n* = 40	Usual care	IG 1: PST, IG 2: Relaxation training	8 healthcare centers and home (IG 2)	Midwife with training and supervision from a clinical psychologist (research personnel)	Postpartum; 6 weeks (6 sessions)	PPD	9 weeks pp	PPD (BDI-II)	Significantly greater decrease in BDI-II in PST and relaxation training groups with greater effect of PST than relaxation
Noorbala et al. (73)	Iran	Cluster RCT	202 pregnant women (6–10 weeks GA); IG *n* = 101, CG *n* = 101	Usual care	Three-tiered intervention for low, medium, and high risk of depression: life skills and stress management training, supportive psychotherapy, educational package, and drug therapies	4 urban healthcare centers	Midwife, physician, and psychiatrist referral for medication management (clinic personnel)	Prenatal and postpartum; Intervention through 6mo pp	Mental health: perinatal depression and anxiety	35–37 weeks GA and 6 weeks and 6 months pp	Depression and anxiety (GHQ-28)	Significantly greater decrease in the IG on GHQ-28 at all assessment points, subscales for somatic complaints and anxiety, but not depression at 6 weeks pp, and subscales for somatic complaints, anxiety, depression, and social function at 6 months pp.
Rahman et al. (79)	Pakistan	Cluster RCT	903 pregnant women (3rd trimester) with depression (DSM-IV structured interview); IG *n* = 463, CG *n* = 440	Enhanced usual care (equal number of home visits by routine Lady Health Workers)	CBT-based manualized intervention for perinatal depression (Thinking Healthy Programme)	Homes within community primary care network	Community health workers (existing personnel)	Prenatal and postpartum; 11 months (16 sessions)	Perinatal depression	6 and 12 months pp	Infant weight and height, Perinatal depression (HDRS)	Significantly greater improvement in IG in HDRS at 6 and 12 months postpartum. No significant difference in infant growth.
Richter et al. (80)	South Africa	Cluster RCT	1200 pregnant women living with HIV; IG *n* = 544, CG *n* = 656	Usual care	Peer mentor group sessions focused on HIV care and encouraging social support development	8 community health or primary healthcare clinics	Peer mentors (recruited for study)	Prenatal and postpartum; 8 sessions	Perinatal depression	1.5 months pp	Depression (GHQ), HIV transmission related behaviors, infant health status post-birth, maternal healthcare utilization, parenting tasks	Significantly greater improvement in IG on GHQ depression score, more likely to ask partners to test for HIV and complete both maternal and infant ARV, but less likely to adhere to ARV during pregnancy.
Rojas et al. (62)	Chile	Individual RCT	230 pp women (within 1 year) with PPD (EPDS > 10 and MINI confirmed); IG *n* = 114, CG *n* = 116	Usual care	Multicomponent: psychoeducational groups, treatment adherence support, structured pharmacotherapy, physician training, specialist supervision	3 urban primary care clinics	Midwives, nurses, physicians (clinic personnel)	Postpartum; 8 weeks (8 sessions)	PPD	3 and 6 months after initiation of intervention	PPD (EPDS)	Significantly greater improvement in IG on EPDS at 3 months, but not difference at 6 months.
Sun et al. (64)	China	Individual RCT	168 pregnant women (12–20 weeks GA) at risk for depression (EPDS > 9 or PHQ-9 > 4); IG *n* = 84, CG *n* = 84	Enhanced usual care (app-based health consultations with a nursing assistant)	Mobile phone application mindfulness training	Home (recruited from hospital perinatal clinic)	Mobile application	Prenatal; 8 weeks	Perinatal depression and anxiety	4, 8, and 18 weeks after initiation of intervention and 6 weeks pp	Perinatal depression (EPDS)	Significantly greater improvement in IG on EPDS, GAD-7, and Positive Affect Schedule
Vakilian et al. (74)	Iran	Individual RCT	44 pregnant women (2nd and 3rd trimester); IG *n* = 22, CG *n* = 22	Usual care	ACT modified to focus on anxiety during pregnancy	5 public health centers	Midwife counseling student supervised by a psychologist (research personnel)	Prenatal; 4 weeks (8 sessions)	Anxiety	Immediate and 1 month post-intervention	Anxiety (PRAQ) and Quality of Life (SF-36)	Significantly greater decrease in IG on PRAQ. No significant difference on SF-36.
Zhao et al. (65)	China	Individual RCT	352 pregnant women (<28 weeks GA) with high-risk pregnancy and at risk for depression (EPDS ≥ 9 or PDSS ≥ 60); IG *n* = 176, CG *n* = 176	Usual care	Couple-separated group psychoeducational program focused on maternal mental health	Obstetrics and gynecology hospital prenatal clinic	Not specified	Prenatal; 6 sessions	Perinatal depression	42 days pp	Psychological status (EPDS, PDSS); Birth outcomes	IG significantly less likely to meet criteria for minor and major depression (EPDS and PDSS), more sleep time, lower cesarean rate, shorter third stage of labor, more satisfaction with family, less concern about caring for infant, less breast milk insufficiency

*EPDS, Edinburgh Postnatal Depression Scale; BDI, Beck’s Depression Inventory; CES-D, Center for Epidemiological Studies Depression Scale; GHQ, General Health Questionnaire; HDRS, Hamilton Depression Rating Scale; PHQ-9, Patient Health Questionnaire-9; PDSS, Postpartum Depression Screening Scale; SCID, Structured Clinical Interview for DSM-IV; STAI, Spielberger’s State-Trait Anxiety Inventory; PRAQ, Pregnancy-Related Anxiety Questionnaire; HAM-A, Hamilton’s Anxiety Rating Scale; PCL-5, PSTD Checklist for DSM-5; PSS, Perceived Stress Scale; FIF, Fatigue Identification Form; SF-36, Short Form Survey-36; AAS, Abuse Assessment Screen; NuPDQ, Revised Prenatal Distress Questionnaire; COPE, Coping Orientation to Problems Experience Inventory; PPD, postpartum depression; PTSD, post-traumatic stress disorder; RCT, randomized controlled trial; pp, postpartum; IG, intervention group; CG, comparison group; GA, gestational age; wk, week; mo, month; yr, year; GBV, gender-based violence; ACT, Acceptance and Commitment Therapy; PST, Problem Solving Therapy; CBT, Cognitive Behavioral Therapy.*

#### Study Design

Five studies were cluster RCTs (73, 78–81) with group assignment at the level of the healthcare center, two were classified as quasi-cluster RCTs (75, 76), and thirteen were individual RCTs (62–72, 74, 77).

#### Study Sample

The sample populations included women in all three trimesters of pregnancy and the postpartum period. Most studies (75%) focused on women in the antepartum period (63–65, 67–69, 71, 73–75, 77–81), while five studies (25%) focused on women in the postpartum period (62, 66, 70, 72, 76). More than half of the studies focused on illness prevention rather than treatment of women with active diagnoses or positive screening for depression or anxiety. Three studies specifically excluded women with elevated Edinburgh Postnatal Depression Screening (EPDS) scores (≥10, >12, and ≥15) (66, 69, 71) and two excluded women with any history of psychiatric disorders (63, 74). Of the 20 studies, three focused on women at risk for depression as determined by depression screening tools (64, 65, 77) and five focused on women with depression confirmed by MINI (Mini-International Neuropsychiatric Interview), CIDI (Composite International Diagnostic Interview), or SCID (Structured Clinical Interview for DSM-IV) (62, 70, 72, 78, 79). One study focused on women with moderate to high anxiety as measured by Spielberger’s State-Trait Anxiety Inventory (STAI) (67). In addition to anxiety and depression status, one study focused on women who reported gender-based violence (75). In addition, four studies focused on special medical populations, including traumatic childbirth (66), high-risk pregnancy (65), and HIV (80, 81).

#### Intervention

**FIGURE 2:**
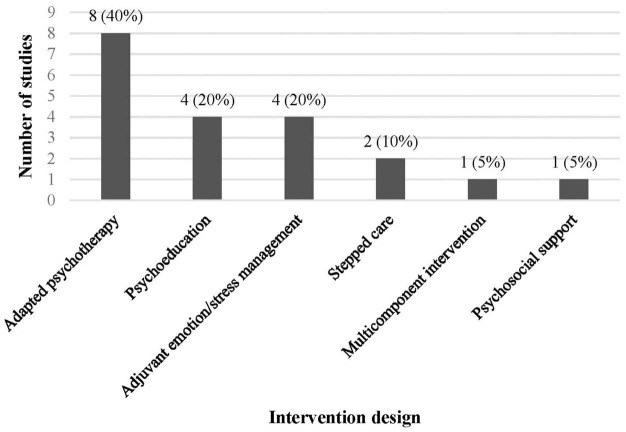
Number and percent of studies by intervention design.

Interventions were variable across studies. The intervention designs included adapted psychotherapies (40%) (63, 66, 68, 70, 72, 74, 79, 81), psychoeducation (20%) (65, 76, 77, 80), adjuvant emotion/stress management techniques (20%) (mindfulness, relaxation, and exercise) (64, 67, 69, 71, 72), stepped care (10%) (73, 78), and a multicomponent intervention (5%) (psychoeducation, pharmacotherapy, and non-specialist training) (62), and psychosocial support (5%) (75) (Figure 2). Four studies included pharmacotherapy as part of the intervention (62, 73, 76, 78). Interventions focused on perinatal (antepartum and postpartum) depression (63, 65, 75, 78–81), postpartum depression only (62, 70–72, 76), anxiety (67, 68, 74), or a combination of perinatal depression and anxiety (64, 66, 69, 73, 77). Interventions were implemented in groups (65, 68, 77, 80), as individuals (64, 66, 67, 69–76, 78, 79, 81), or a combination of both (62, 63). Fifty percent of interventions were conducted in the antepartum period (63–65, 67–69, 74, 75, 77, 81), 25% in the postpartum period (62, 66, 70, 72, 76) and 25% spanning across the antepartum and postpartum period (71, 73, 78–80). Intervention duration included anywhere from 1 single session to 16 session over 11 months, with the most interventions conducted over 3–8 weeks.

In most studies (65%) the intervention was conducted completely within a healthcare center, including community and public health centers, primary care clinics, maternal and child health centers, and perinatal clinics (62, 63, 65, 67, 68, 73–78, 80, 81). Two studies recruited participants from healthcare centers and the intervention was completed on a mobile application outside of the healthcare center (64, 70). Three of the adjuvant interventions were completed both in healthcare centers and at home (69, 71, 72) and one adapted psychotherapy intervention had sessions conducted in the healthcare center and over the phone (66). The final study intervention was conducted only within homes in the context that healthcare within rural regions of the country was routinely delivered in home settings by community healthcare workers that were part of primary healthcare systems (79).

Intervention personnel were variable across studies and included nurses (62, 76), community health workers (79), midwives (62, 66, 72–74), peer mentors (80, 81), obstetricians or primary care providers (62, 63, 73, 78), and mental health professionals (psychotherapists or psychiatrists) (68, 73). Eight studies indicated utilizing research personnel or recruited peer mentors specifically for intervention implementation (63, 66, 68, 72, 74–76, 80). Four studies included referrals to mental health professionals if needed (72, 73, 75, 78). Seven studies reported ongoing supervision of non-mental health professionals and intervention personnel beyond initial intervention training (62, 66, 72, 74, 78–80).

#### Assessment and Outcomes

Primary outcomes were as per the inclusion criteria (change in depression and/or anxiety scores), and outcome measures varied greatly across studies. Eight different depression screening instruments were utilized, the EPDS was the most frequent (59% of studies that focused on depressive symptoms) (62–66, 69–71, 75, 78). Other measures of depression included the BDI (Beck’s Depression Inventory) (72, 76, 77), CES-D (Center for Epidemiological Studies Depression Scale) (81), GHQ (General Health Questionnaire) (73, 80), HDRS (Hamilton Depression Rating Scale) (79), PHQ-9 (Patient Health Questionnaire-9) (63), PDSS (Postpartum Depression Screening Scale) (65) and SCID (63, 77). Anxiety measures included the GHQ (73), STAI (67–69), PRAQ (Pregnancy-Related Anxiety Questionnaire) (74), HAM-A (Hamilton’s Anxiety Rating Scale) (66), and one PTSD measure: PCL-5 (PSTD Checklist for DSM-5) (66). Assessment of outcomes varied greatly across studies, ranging from immediately post-intervention to 12 months postpartum. Nine studies had one assessment point (65, 67, 68, 70, 72, 75, 76, 80, 81) and 11 studies had more than one assessment point (62–64, 66, 69, 71, 73, 74, 77–79).

Nearly all studies demonstrated significantly greater improvement in depression and/or anxiety measurement scores in the intervention group(s) as compared to the control group (Table 1). Three exceptions are noted. One study assessing an exercise intervention showed no significant difference in EPDS scores (71). Another study examining a group manualized psychoeducational intervention in women at high-risk for depression demonstrated significant reduction on the BDI-II in both intervention and control groups and no significant treatment effect, but found significantly fewer new depression cases in the intervention group per the SCID (77). The third study was a multicomponent intervention that demonstrated significantly greater improvement in the EPDS in the intervention group at 3 months, but significant differences were not maintained at 6 months (62).

### Assessment of Risk of Bias

Quality scores based on Downs and Black criteria (61) ranged from 68 to 96% with a mean score of 80%. Categories with lower quality ratings included lack of reporting of adverse events (90%), inability to blind study subjects to intervention (55%), not taking into account losses to follow-up (45%) and non-compliance with the intervention (35%) (Supplementary Table 1).

## Discussion

Integrating perinatal mental health care into routine maternal care is an important strategy for improving access to mental health care in under-resourced LMICs. However, there are a substantial lack of resources and differences in health care systems, culture, and language in LMICs as compared to HIC, where these interventions were developed. As such, it is critical to understand intervention effectiveness in LMICs prior to intervention implementation and scale-up. This is the first systematic review examining the effectiveness of interventions that integrate perinatal mental health care into routine maternal care in LMICs.

Overall, the present findings demonstrate that interventions that integrate perinatal mental health care into routine maternal care can be effective in LMICs for women with depression and anxiety. That is, nearly all studies demonstrated significantly greater improvement on depression and/or anxiety measures in the intervention group(s) as compared to controls. However, the substantial heterogeneity between the included studies limits interpretation, comparison, and generalizability of the data. Nonetheless, the findings provide a critical understanding of the gaps in current integrated intervention strategies and essential insights into methods to overcome barriers to integrated care design and implementation in LMICs. These findings provide a valuable foundation for future evidence-based adaptation, implementation, and scale-up of interventions that integrate perinatal depression and anxiety care into routine maternal care in LMICs.

The heterogeneity between the included studies is most notable among the study sample selection, intervention designs, and outcome assessments. Study samples were quite variable across studies: 55% of the included studies carried out preventive strategies and did not focus on women with active diagnoses or who screened positive for depression or anxiety. This included five studies that actively excluded women who had elevated screening scores for depression (≥10, >12, and ≥15) or had any history of psychiatric disorders (63, 66, 69, 71, 74). Alternatively, the remaining studies took a treatment approach and selectively focused on women at risk for depression as determined by screening tools (64, 65, 77), women with confirmed depression by structured diagnostic interviews (62, 70, 72, 78, 79), and women with moderate to high anxiety on screening (67).

The heterogeneity between study intervention designs provides valuable insight into the state of interventions that integrate perinatal depression and anxiety care into routine maternal care in LMICs. Most studies (85%) implemented and evaluated interventions that employed psychosocial, psychoeducational, psychotherapeutic, or adjuvant emotion/stress management strategies (Figure 2). Two studies (10%) utilized a stepped care approach to integrated care (73, 78) and one study (5%) implemented a multicomponent approach comprising psychoeducation, pharmacotherapy, and non-mental health professional training and specialist supervision (62). Further heterogeneity was seen in intervention design in which only four studies incorporated the potential need for pharmacotherapy within their interventions and only four studies included referrals to mental health professionals, if needed. Additional variability included differences in group vs. individual implementation, implementation personnel (including peer mentors, community health workers, midwives, obstetricians, primary care providers, and mental health professionals), duration of interventions, and location of intervention implementation (healthcare centers vs. home).

Finally, heterogeneity of outcome assessment is highlighted by the variety of outcome measures utilized among the studies. Although 59% of studies that were focused on depression utilized the EPDS, the remaining studies utilized seven other measures of depression symptoms and five different measures of anxiety symptoms. The number of assessment time points and timing of outcomes assessment was also highly variable across studies which limits both study comparison and the conclusions that can be drawn, particularly regarding sustained and long-term effectiveness of interventions. Many studies did not complete follow-up into the postpartum period and of those that did, very few completed assessment beyond 6 months postpartum.

While the heterogeneity of the study sample selection, intervention designs, and outcome assessments limits study comparison, this does not take away from the overall evidence that integrated interventions can be effective in LMICs. Nearly all studies reported significantly greater improvement on the primary outcomes (depression and/or anxiety measures) in the intervention group(s) as compared to control. The only study that found no significant differences was an adjuvant home exercise intervention with reported poor adherence (71). Two additional studies demonstrated mixed results. One reported significantly fewer new depression cases on diagnostic interview (SCID) in the intervention group, but found no difference in depression screening scores on the BDI-II as compared to the control group (77). The second study demonstrated significantly greater improvement on depression screening on the EPDS 3 months after intervention initiation, but the difference was not sustained at the 6 months assessment (62). Nonetheless, the interpretation of study outcomes is limited by the lack of included studies describing the translation, adaptation, and validation of measures to the local setting. This is an essential component to outcome measurement in LMICs given the substantial differences in culture and language that can impact outcome measure validity. Ultimately, it limits our understanding of the included studies and interventions.

The findings highlight gaps in the interventions that integrate perinatal depression and anxiety care into routine maternal care. First, more than half of the included study interventions focused on prevention and did not aim to address women with positive screening or diagnosis of depression and anxiety, particularly moderate to severe symptoms. This raises the concern that women with moderate to severe symptoms and at the highest risk of adverse outcomes are not being addressed within current interventions. It also highlights the dilemma of where limited LMIC resources should be directed in addressing perinatal depression and anxiety. Secondly, interventions were mostly conducted either in the antepartum period or the postpartum period and very few covered both time periods. This introduces a risk of perinatal depression or anxiety going undetected and untreated. Finally, while all included study interventions aimed to integrate perinatal mental health care into routine maternal care, 85% focused on only single intervention designs, specifically, psychological, psychosocial, psychoeducational, or adjuvant emotion/stress management interventions. There were very few multicomponent approaches and interventions that incorporated pharmacotherapy, ongoing non-mental health professional supervision, and referral to mental health specialists.

The lack of multicomponent integrated interventions highlights an important gap in LMICs: the crucial need for implementation and assessment of comprehensive interventions that address the needs of women along the entire spectrum of illness. In fact, the WHO recommendation to integrate perinatal mental health care into routine maternal care specifically outlines a comprehensive approach with increasing interventions based on illness severity. This includes (1) monitoring and increasing support based on individual needs, (2) psychosocial support, (3) psychoeducation, (4) evidence-based adapted psychotherapies, (5) training and (6) supervision of health care workers, (7) referral to specialist mental health providers, (8) prescription of psychotropic medications, and (9) consideration of special programs for unique populations, such as women living with HIV or experiencing family violence (37).

Review of the included study interventions within the WHO recommended comprehensive approach framework further emphasizes the limitations of these interventions (Table 2). The average number of components included in study interventions was 2.8 (±2.1) out of 9. The study interventions that addressed the greatest number of these recommendations were the stepped care and multicomponent interventions (five to seven components). In fact, these three interventions were the only ones to address the WHO recommended approach of increasing intensity of interventions based on illness severity.

**TABLE 2:** Intervention components of included studies by WHO recommended intervention component (37).

Study	Increasing interventions based on illness severity	Psychosocial support	Psychoe- ducation	Adapted psychotherapy	Mental health specialist referral	Pharma- cotherapy	Non-mental health professional training	Non-mental health professional ongoing supervision	Unique populations
Asadzadeh et al. (66)				X			X	X	X
Bastani et al. (67)							X		
Esfandiari et al. (68)				X					
Futterman et al. (81)		X		X			X		X
Gureje et al. (78)	X	X		X	X	X	X	X	
Jabbari et al. (69)									
Jannati et al. (70)				X					
Kariuki et al. (76)			X			X	X		
Lara et al. (77)			X						
Mao et al. (63)				X			X		
Mohammadi et al. (71)									
Mutisya et al. (75)		X			X		X		X
Nasiri et al. (72)				X	X		X	X	
Noorbala et al. (73)	X	X		X	X	X	X		
Rahman et al. (79)				X			X	X	
Richter et al. (80)		X	X				X	X	X
Rojas et al. (62)	X		X			X	X	X	
Sun et al. (64)									
Vakilian et al. (74)				X			X	X	
Zhao et al. (65)			X						

Future integrated intervention design in LMICs will benefit from adapting care models that utilize comprehensive approaches with increasing intensity of care components depending on patients’ needs. One such example from the included studies is the stepped care approach. Stepped care is an evidence-based integrated care model designed to be the least resource-intensive by providing increasing intensity of care to patients depending on illness severity (82, 83). While this model is designed to be the least resource-intensive, it will likely still prove challenging to implement within the constraints of substantially under-resourced LMIC health care settings. However, as demonstrated in the included studies, it offers a versatile approach that can be adapted to match local resources. This includes referral to higher levels of specialized care, which may prove more feasible for managing more severe illness in highly resource limited primary care settings. Models such as stepped care designs also offer a more sustainable approach to mental health care in LMICs through a focus on training and supervising existing non-mental health professionals. This is a notable advantage, as several of the included studies with single component interventions relied on research personnel to carry out the interventions rather than training existing personnel (Table 1). Additionally, several studies also noted poor adherence to single component interventions, particularly multi-session adapted psychotherapy and psychoeducation interventions. Alternatively, multicomponent interventions could provide more variable opportunities for engaging patients in care. Nonetheless, while more comprehensive integrated care models can address a greater spectrum of illness and offer more opportunities to engage patients, they require more resource commitment and can place an additional burden on existing personnel. As such, it is necessary to examine each setting individually and adapt interventions to fit with the local resources, as well as ensure that the benefits to patients and providers outweigh any additional burden.

The current findings also provide critical insights for overcoming barriers to designing and implementing interventions that integrate perinatal mental health care into routine maternal care in LMICs. This includes the need for adaptation to the local health care setting, culture, language, and available resources. First, the included studies highlight the need for intervention adaptation to local health care settings. Health care systems can be quite variable across LMICs, in addition to differences as compared to HICs. For instance, urban and rural healthcare in LMICs can vary dramatically, ranging from specialty maternal clinics for high-risk pregnancies to primary maternal care provided in patient’s homes by community health workers. The included studies demonstrate adaptation of intervention design to their local healthcare systems, such as adapting complexity of interventions to the training level of local personnel.

Secondly, the present findings highlight intervention adaptation that leverages locally available resources, such as providing interventions in group formats, utilizing peer mentors and community health workers, developing mobile application-based interventions, and integrating mental health interventions into already existing social support programs for pregnant women. Thirdly, the included studies highlight the importance of intervention adaptation that also addresses the psychosocial risk factors that place women in LMICs at greater risk for perinatal mental illness. For instance, some programs focus on addressing gender-based violence, developing social support networks, involving partners, and guiding patients in obtaining government economic support. Lastly, while the variability in outcome measures makes comparison among studies challenging. It highlights the essential need for cultural and language adaptation of intervention and assessment, such that outcome measures in LMICs is often chosen based on previously translated, adapted, and validated measures in the local setting. The necessity of cultural and language adaptation for success of integrated interventions in LMICs cannot be understated.

These insights for the design and implementation of integrated interventions in LMICs are beneficial not only for future implementation and scale-up of interventions in LMICs, but also provide strategies for reverse integration in under-resourced settings in HICs. For instance, HIC integrated programs could benefit from further addressing psychosocial risk factors, integrating the use of mobile applications, developing home and community-based interventions, and utilizing alternative methods of care delivering, including group interventions, peer mentorship, and community health workers.

There are important limitations to consider in interpreting the present findings. The heterogeneity of the included studies limits the overall comparison of studies, the conclusions that can be drawn regarding the effectiveness of integrated interventions, and the overall generalizability of the data. The substantial study heterogeneity specifically limited the ability to complete a meta-analytic assessment, limiting the overall conclusions that can be drawn regarding intervention effectiveness. Furthermore, the lack of included studies conducted in low-income countries and the remaining 20 studies representing only eight middle-income countries is notable and limits the overall generalizability of the findings for low-income countries. It also highlights the inequities of mental health research conducted in LMICs (36). Additionally, while the inclusion criteria selection of only controlled trials provides more robust data, it also means that it is not a full representation of all interventions that have integrated perinatal depression and anxiety care into routine maternal care in LMICs. During full text review, additional articles describing integrated care interventions were ultimately excluded due to lack of a comparison group or lack of outcome measures (84–88). However, the inclusion criteria selection for controlled trials over RCTs did allow for greater study inclusion and representation of existing interventions, as was intended. Nonetheless, this highlights the need for more robust structured assessment of integrated interventions prior to wider implementation in LMICs.

In conclusion, the findings demonstrate that interventions that integrate perinatal mental health care into routine maternal care for women with depression and anxiety can be effective in LMICs. However, the substantial heterogeneity between studies limits the conclusions that can be drawn and generalizability. Nonetheless, the findings provide a critical understanding of the gaps in current integrated interventions in LMICs. This includes the lack of comprehensive intervention designs that incorporate increasing intensity of treatment for more severe illness, pharmacotherapy, mental health specialist referrals, and non-mental health professional training and ongoing supervision. Study findings also provide strategies to overcome design and implementation barriers in LMICs, particularly methods for successful adaptation to the local health care setting, available resources, psychosocial challenges, and local culture. These findings provide a foundation for future evidence-based adaptation, implementation, and scale-up of interventions that integrate perinatal mental health care into routine maternal care in LMICs. Additionally, the findings provide strategies for reverse integration for improving HIC approaches in under-resourced settings and diverse patient populations.

## Data Availability Statement

The original contributions presented in the study are included in the article/Supplementary Material, further inquiries can be directed to the corresponding author/s.

## Author Contributions

LP and MP designed the electronic database search strategy and LP completed the search. MP and AD completed abstract and full text article reviews, data extraction, and wrote the first draft of the manuscript. All authors contributed to conception and design of the systematic review and contributed to manuscript revision, read, and approved the submitted version.

## Conflict of Interest

NB received honoraria from Global Learning Collaborative, Medscape, and Mathematica. She has served as a consultant for The Kinetix Group. The remaining authors declare that the research was conducted in the absence of any commercial or financial relationships that could be construed as a potential conflict of interest.

## Publisher’s Note

All claims expressed in this article are solely those of the authors and do not necessarily represent those of their affiliated organizations, or those of the publisher, the editors and the reviewers. Any product that may be evaluated in this article, or claim that may be made by its manufacturer, is not guaranteed or endorsed by the publisher.
