# Supplementary material for: A Systematic Review of Interventions That Integrate Perinatal Mental Health Care Into Routine Maternal Care in Low- and Middle-Income Countries
Source: Front Psychiatry. 2022 Mar 14;13:859341. doi: 10.3389/fpsyt.2022.859341 (PMC8964099; doi:10.3389/fpsyt.2022.859341)
Supplement: Supplementary file 1 [file Table_1.pdf]

Supplementary Table 1: Downs and Black Quality Assessment Scoring

	Checklist Question																											
	Reporting										External Validity			Internal Validity (bias)							Internal Validity (confounding)						Power	
**Study**	**1**	**2**	**3**	**4**	**5**	**6**	**7**	**8**	**9**	**10**	**11**	**12**	**13**	**14**	**15**	**16**	**17**	**18**	**19**	**20**	**21**	**22**	**23**	**24**	**25**	**26**	**27***	**TOTAL**
Asadzadeh et al, 2020 (66)	1	1	1	1	2	1	1	0	1	0	1	0	1	0	1	1	1	1	1	1	1	1	1	1	1	1	1	**24**
Bastani et al, 2005 (67)	1	1	1	1	2	1	1	0	1	1	1	0	1	0	0	1	1	1	0	1	1	1	1	1	0	0	1	**21**
Esfandiari et al, 2020 (68)	1	1	1	1	2	1	1	0	1	1	1	0	1	0	1	1	1	1	1	1	1	1	1	1	1	1	1	**25**
Futterman et al, 2010 (81)	1	1	0	1	2	1	1	0	1	1	1	0	1	0	0	1	1	1	1	1	1	1	0	0	1	1	0	**20**
Gureje et al, 2019 (78)	1	1	1	1	2	1	1	1	1	1	1	0	1	1	1	1	1	1	1	1	1	1	1	1	1	1	1	**27**
Jabbari et al, 2020 (69)	1	1	1	1	2	1	1	0	1	0	1	0	1	1	1	1	1	1	0	1	1	1	1	1	0	1	1	**23**
Jannati et al, 2020 (70)	1	1	1	1	2	1	1	0	1	1	1	0	1	0	0	1	1	1	0	1	1	1	1	1	0	1	1	**22**
Kariuki et al, 2021 (76)	1	1	1	1	2	1	1	0	1	1	0	0	1	0	1	1	1	1	0	1	1	1	1	0	1	1	1	**22**
Lara et al, 2010 (77)	1	1	1	1	2	1	1	0	0	1	0	0	1	0	0	1	1	1	1	1	1	1	0	0	1	0	1	**19**
Mao et al, 2012 (63)	1	1	1	1	2	1	0	0	1	1	1	0	1	0	1	0	1	1	1	1	1	0	1	1	1	1	0	**21**
Mohammadi et al, 2015 (71)	1	1	1	1	2	1	1	0	0	1	0	0	1	1	1	1	1	1	1	1	1	1	1	1	1	0	0	**22**
Mutisya et al, 2017	1	1	1	1	2	1	1	0	0	1	1	0	1	1	1	1	1	1	0	1	1	0	1	1	1	0	1	**22**
Nasiri et al, 2018 (72)	1	1	1	1	2	1	1	1	0	1	1	0	1	0	0	1	1	1	1	1	1	1	1	1	1	0	0	**22**
Noorbala et al, 2019 (73)	1	1	1	1	2	1	1	0	0	1	1	0	1	1	0	0	1	1	1	1	1	1	1	0	1	0	0	**20**
Rahman et al, 2008 (79)	1	1	1	1	2	1	1	0	0	1	1	0	1	1	1	1	1	1	1	1	1	1	1	1	1	0	1	**24**
Richter et al, 2014 (80)	1	1	0	1	2	1	1	0	1	1	1	0	1	1	0	1	0	1	1	1	1	1	1	0	1	0	1	**21**
Rojas et al, 2007 (62)	1	1	0	1	2	1	1	0	1	1	1	0	1	1	1	1	1	1	0	1	1	1	1	1	1	1	1	**24**
Sun et al, 2021 (64)	1	1	1	1	2	1	1	0	1	1	1	0	1	1	1	1	1	1	0	1	1	1	1	1	1	1	1	**25**
Vakilian et al, 2019 (74)	1	1	1	1	2	1	1	0	1	1	1	0	1	0	0	0	1	1	1	1	1	1	1	1	1	1	1	**23**
Zhao et al, 2017 (65)	1	1	1	1	2	1	0	0	0	1	1	0	1	0	0	1	1	1	1	1	1	1	1	1	0	0	1	**20**

*Item 27, a five-point item evaluating sample size and power, was dichotomized to indicate if the study reported a priori power and sample size calculations, for an adjusted maximum (total) score of 28. See Downs and Black for full list of criteria (61).
